# Development and validation of a diagnostic model for early differentiation of sepsis and non-infectious SIRS in critically ill children - a data-driven approach using machine-learning algorithms

**DOI:** 10.1186/s12887-018-1082-2

**Published:** 2018-03-15

**Authors:** Florian Lamping, Thomas Jack, Nicole Rübsamen, Michael Sasse, Philipp Beerbaum, Rafael T. Mikolajczyk, Martin Boehne, André Karch

**Affiliations:** 1 Department of Epidemiology, Research Group Epidemiological and Statistical Methods (ESME), Helmholtz Centre for Infection Research, Inhoffenstr. 7, 38124 Braunschweig, Germany; 2 Department for Pediatric Cardiology and Intensive Care Medicine, Hannover Medical School, 30625 Hannover, Germany; 3 German Center for Infection Research (DZIF), Hannover-Braunschweig site, 30625 Hannover, Germany

**Keywords:** Diagnosis, Sepsis, SIRS, Pediatric, Random Forest, Intensive care unit

## Abstract

**Background:**

Since early antimicrobial therapy is mandatory in septic patients, immediate diagnosis and distinction from non-infectious SIRS is essential but hampered by the similarity of symptoms between both entities. We aimed to develop a diagnostic model for differentiation of sepsis and non-infectious SIRS in critically ill children based on routinely available parameters (baseline characteristics, clinical/laboratory parameters, technical/medical support).

**Methods:**

This is a secondary analysis of a randomized controlled trial conducted at a German tertiary-care pediatric intensive care unit (PICU). Two hundred thirty-eight cases of non-infectious SIRS and 58 cases of sepsis (as defined by IPSCC criteria) were included. We applied a Random Forest approach to identify the best set of predictors out of 44 variables measured at the day of onset of the disease. The developed diagnostic model was validated in a temporal split-sample approach.

**Results:**

A model including four clinical (length of PICU stay until onset of non-infectious SIRS/sepsis, central line, core temperature, number of non-infectious SIRS/sepsis episodes prior to diagnosis) and four laboratory parameters (interleukin-6, platelet count, procalcitonin, CRP) was identified in the training dataset. Validation in the test dataset revealed an AUC of 0.78 (95% CI: 0.70–0.87). Our model was superior to previously proposed biomarkers such as CRP, interleukin-6, procalcitonin or a combination of CRP and procalcitonin (maximum AUC = 0.63; 95% CI: 0.52–0.74). When aiming at a complete identification of sepsis cases (100%; 95% CI: 87–100%), 28% (95% CI: 20–38%) of non-infectious SIRS cases were assorted correctly.

**Conclusions:**

Our approach allows early recognition of sepsis with an accuracy superior to previously described biomarkers, and could potentially reduce antibiotic use by 30% in non-infectious SIRS cases. External validation studies are necessary to confirm the generalizability of our approach across populations and treatment practices.

**Trial registration:**

ClinicalTrials.gov number: NCT00209768; registration date: September 21, 2005.

**Electronic supplementary material:**

The online version of this article (10.1186/s12887-018-1082-2) contains supplementary material, which is available to authorized users.

## Background

Sepsis and the systemic inflammatory response syndrome (SIRS) are two conditions with similar pathophysiological patterns and symptoms, but different causes of disease [1–3]. While the systemic immune response in sepsis is caused by pathogens, non-infectious SIRS is due to non-infectious triggers. In children, sepsis is defined as the presence of SIRS during evidence of an infection [1, 3]. Evidence for an infection is typically provided by pathogen identification in the blood (mainly by blood culture analyses), or by presence of clinical symptoms associated with a high probability of systemic infection [1–4]. However, blood culture sampling often yields false-negative results, and clinical signs of infection are often unspecific. It is therefore a huge challenge to diagnose sepsis correctly in early disease states, which would be necessary to initiate prompt antimicrobial treatment and to reduce case fatality rates [5]. Therefore, many patients with fulfilled SIRS criteria but weak evidence of infection are unnecessarily treated with antimicrobial agents. This may be associated with adverse drug effects, favor the emergence of multi-resistant bacteria and increase healthcare costs [6].

In the past decades, several biomarkers have been proposed as diagnostic tests for the differentiation of sepsis and non-infectious SIRS [7, 8], like e.g. procalcitonin (PCT) and interleukin-6 (IL-6) [9–11]. However, none of them was considered suitable to diagnose sepsis with sufficient accuracy in clinical practice [12]. In some cases, initial study results were overoptimistic due to flawed study designs and lack of external validation [10, 11]; in others, the proposed markers were too expensive or too difficult to obtain for being implemented in the therapeutic standards of intensive care medicine [13]. In an adult population, a recent study showed that the discriminatory ability of several weak sepsis biomarkers could be improved when combining them into one diagnostic model [14]. However, even this combination could not sufficiently improve the accuracy for sepsis/non-infectious SIRS discrimination [14, 15]. Due to age-related changes in symptoms and laboratory markers, diagnosis of sepsis and distinction from non-infectious SIRS are even more complex in children.

Our aim was to develop and validate a diagnostic model for the discrimination of pediatric sepsis and non-infectious SIRS during the clinical course based on routinely available parameters, which can easily be implemented into clinical practice. Therefore, we decided to perform a fully data-driven approach using all information gathered on a pediatric intensive care unit (PICU) during a randomized clinical trial (RCT) with a homogeneous and validated definition for sepsis and non-infectious SIRS.

## Methods

### Source of data

Data used for this analysis arise from a prospective single-center RCT investigating the effect of in-line filtration in an interdisciplinary PICU of a German tertiary care hospital (ClinicalTrials.gov number: NCT00209768) [16]. Patient recruitment took place between February 2005 and September 2008.

### Outcome

Outcome of interest was the presence of non-infectious SIRS or sepsis according to the criteria defined by the international pediatric sepsis consensus conference (IPSCC) in 2005 [1, 3]. Sepsis was diagnosed according to IPSCC criteria as “SIRS in the presence of or as a result of suspected or proven infection”. To further improve the correctness and validity of the infectious origin we additionally applied the consensus conference criteria for infection in the intensive care unit [17]. All sepsis diagnoses were later reviewed according to the updated Centers for Disease Control and Prevention (CDC) criteria from 2008 [18] as indicated. A catheter-related sepsis with common skin commensals as coagulase negative staphylococci was defined according to the consensus conference criteria for infection in the intensive care unit [3]. Further information about all sepsis episodes including the sites of primary infection as well as microbiological test results can be found in the additional files (Additional file 1: Table S1).

Diagnoses of SIRS/sepsis were made prospectively in real-time by an experienced attending physician with the consultation of infectious disease specialists. The diagnoses were later reviewed independently by two blinded experienced pediatric intensive care physicians. The confirmatory review was a post-hoc analysis with the availability of all clinical data such as vital signs, infectiological, laboratory and radiological data. This final analysis was performed after discharge of the patient from PICU and after checks for data integrity and validity. In case of disagreement, a consensus was achieved after open discussion with a third senior pediatric intensive care physician and the episode was allocated without ambiguity to either non-infectious SIRS or sepsis. The reviewers initiated the original study, but were not involved in the data analysis concept of the present analysis.

### Study participants

**Fig. 1:**
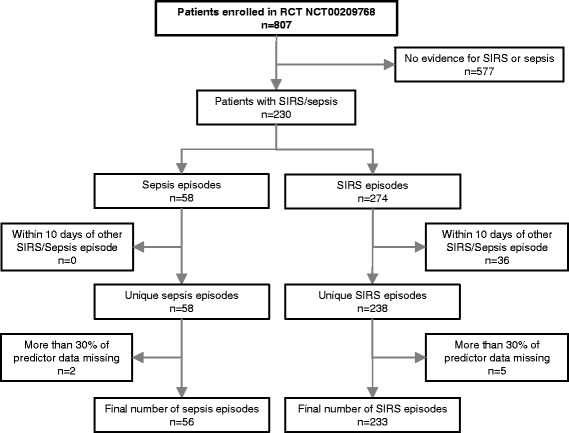
Flow diagram showing the selection criteria for included non-infectious SIRS and sepsis episodes. Sepsis and non-infectious SIRS were discriminated according to the International Pediatric Sepsis Consensus Conference (IPSCC) criteria [1, 3], and were confirmed by two blinded experienced pediatric intensive care physicians. Each episode of disease was assigned to either non-infectious SIRS or sepsis without ambiguity

All patients under the age of 18 years admitted to the PICU were eligible for enrollment in the original RCT. Exclusion criteria covered expected death within 48 h of admission, participation in other trials, or absence of intravenous therapy. Individual follow-up began at enrollment and ended with discharge from the PICU, death, or discontinuation of allocated interventional therapy. Discharge within 6 h after admission was a reason for exclusion from the study [16]. Eight hundred seven patients formed the final dataset of the original RCT. Only patients who developed non-infectious SIRS or sepsis during their ICU stay were considered for the analysis. The total number of diagnosed non-infectious SIRS and sepsis episodes was 274 and 58, respectively. These episodes occurred in 230 patients (Fig. 1); 213 had at least one non-infectious SIRS episode, 47 at least one sepsis episode; 20 suffered from both non-infectious SIRS and sepsis. In order to avoid bias towards disease types occurring early during PICU visit (e.g. post-surgery SIRS), we included not only the first, but all non-infectious SIRS and sepsis episodes of a patient into our analysis. However, we considered only episodes for inclusion, which were diagnosed at least 10 days after termination of the previous episode to avoid any effect of the prior episode on parameter measures. Thus, the primary dataset of our study included 238 non-infectious SIRS and 58 sepsis episodes (Fig. 1).

### Predictors

Forty-six variables were considered as potential predictors in the development stage of the model (Additional file 1: Table S2). All predictor values were extracted from the trial database and were based on parameters obtained from the hospital information system or from patient records. For time-dependent predictors only values at the day of diagnosis were considered (before start of treatment). If more than one value per day was measured for a predictor, the most abnormal value was recorded. All parameter values were checked for plausibility first by the responsible clinicians and statisticians of the original RCT, and again by the statisticians of this secondary analysis. Continuous predictor variables were kept continuous. If age- and sex-specific reference values were available, we standardized the respective parameters for age and sex (Additional file 1: Table S2) by dividing the measured value by the mean reference value of the respective age group.

### Missing data

Missing data were handled in a three-step approach based on a missing at random assumption. First, if a value for a given predictor was missing but there were values on the day before and on the day after the event, the arithmetic mean of these two values was used for imputing the missing value. In a second step, all predictors containing more than 30% missing values, and all episodes which were associated with missing values in more than 30% of the predictors considered were excluded since missForest (the imputation method used subsequently) provides unbiased imputation results for up to 30% missing values [19, 20]. After application of exclusion criteria related to missing values, two variables (central venous oxygen saturation and glutamate dehydrogenase) as well as five non-infectious SIRS and two sepsis episodes were excluded, resulting in a final dataset of 233 non-infectious SIRS and 56 sepsis episodes (Fig. 1) and 44 variables.

All other missing values were imputed using the R package missForest (version 1.4, [19, 20]). MissForest is a nonparametric missing value imputation methodology able to handle mixed-type data [19]. It was shown to outperform other widely used imputation techniques, such as multivariate imputation by chained equations (MICE) and k nearest neighbour imputation (KNNimpute), especially when complex interactions and nonlinear relations are suspected as it was the case with our dataset [19, 20]. Imputation was done leaving out the outcome variable as well as the variables counting the previous events (see Additional file 1: Table S2). Imputation with missForest was performed independently for training and test datasets. The variable “base excess” was excluded after imputation since it represented a linear combination of variables already present in the dataset.

### Statistical analyses

#### Methodological concept

Machine learning is a branch of artificial intelligence used for data analysis which automates analytic model building. Random forests are a method typically used for classification problems which uses machine learning algorithms. Due to the high-dimensional data and the unclear predictor structure, we chose a random forest (RF) approach [21–23] based on conditional inference trees [24] for analysis. While classic statistical modelling techniques building on regression methodology cannot be used in cases where the number of potential predictors exceeds the number of observations, Random Forests have been shown to perform well in these situations [23]. Our analysis approach was data driven since we did not make any a-priori judgements about what kind of variables to use as potential predictors or about what kind of distributions the respective variables might follow. Predictor selection was performed using a backward selection process based on out-of-bag areas under the curve (OOB-AUC [25]). This approach is known to give the same weight to both occurring classes irrespective of the class size [25, 26]. We used the recently developed AUC-based permutation Variable Importance Measure (VIM) [26] which has been shown to be the best selection method in the case of imbalanced datasets as present in our analysis [26]. The model with the largest OOB-AUC was selected as the model of choice. No penalization for the number of selected variables was applied since AUCs were already calculated based on internal validation minimizing the risk of overfitting. A more detailed description of the methodological concept can be found in Additional file 1: Methods S1.

#### Statistical software

All analyses were performed using the R package party, version 1.0–22 [26]. By setting the parameters mincriterion, minbucket and minsplit in the cforest function to zero, conditional inference trees were grown to maximal possible depth [26]; bootstrap sampling was used as the resampling scheme; the number of trees per forest was set to 1000. The mtry parameter was set to the square root of the number of predictor variables. All parameters were hold fixed throughout the entire analysis. R codes used for this analysis are presented in Additional file 1: Code S1.

#### Model validation

The dataset was split into two parts (training and validation dataset) in a non-random manner. Patients enrolled 2005–2006 were used for the training dataset, while those enrolled in 2007–2008 served as the validation dataset. Non-random time splits represent one of the best validation methods when no truly external validation dataset is available and provide considerably more valid results than random splits of datasets; they are therefore considered an intermediate between internal and external validation [27]. Areas under the curve (AUCs) with DeLong confidence intervals were used as a measure of diagnostic accuracy. Sensitivity and specificity of sepsis diagnosis (with respective Wilson confidence intervals) were calculated for two cut-off values defined by a) the Youden index [28] and b) the lowest cut-off probability associated with 100% correct classification rate for sepsis.

#### Comparison to previously proposed individual markers

We evaluated the diagnostic accuracy of previously proposed markers for differentiation of non-infectious SIRS and sepsis (C-reactive protein [CRP], PCT, IL-6) and their combination in our validation dataset and compared it to the accuracy of the diagnostic model developed in the RF approach.

#### Sensitivity analyses

For sensitivity analyses, we first varied the mtry parameter of the RF procedure for our primary analysis to estimate the stability of our methodological concept. Second, we assessed the stability of the validation concept used for our primary analysis by comparing it to a three-fold internal cross-validation approach. Cross-validation (CV) is a widely used resampling method in machine learning to assess model performance [29]. Thereby the data is split into different parts or folds. Often 3-fold, 5-fold, 7-fold or even 10-fold CV is used. In the case of 3-fold CV the model is built on two folds of the data and model performance is assessed on the other fold of the data. This procedure is than repeated three times so that every fold is once used as test data to assess model performance. Therewith 3 performances measures are obtained which are usually averaged to get the average CV-AUC. We followed this principle and applied our entire data analysis approach (including missing data imputation with MissForest and variable selection) each time to two folds of the data and used the third fold as an independent test data to assess model performance. Third, we ran a sensitivity analysis limiting the study population to one episode per patient (randomly drawn). Fourth, we developed a prediction model using the entire dataset for both training and testing to show how the predictive performance would be overestimated if internal validation was lacking. This can be understood as a bad practice example to show how previous studies might have overestimated the true predictive performance of their models.

## Results

### Study participants

Sepsis episodes were more likely to occur in patients with higher PIM-II score (*p* = 0.034), longer duration of PICU stay until onset of disease (*p* <  0.001), previous history of SIRS and/or sepsis (*p* <  0.001), and were associated with higher levels of PTT (*p* = 0.013), d-dimers (*p* = 0.001), fibrinogen (*p* = 0.018), IL-6 (*p* = 0.001), PCT (*p* = 0.020), CRP (*p* = 0.009), body temperature (*p* <  0.001) and lower levels of platelets (*p* = 0.023). In the blood gas analysis, sepsis episodes showed higher bicarbonate (*p* = 0.048), whereas SpO_2_ (*p* = 0.015) values were lower in sepsis than in non-infectious SIRS episodes (Table 1).

**Table 1:** Patient characteristics stratified by non-infectious SIRS/sepsis (*n* = 289)

Predictor variable	Sepsis (*n* = 56) frequency/median (1st quartile-3rd quartile)	Non-infectious SIRS (*n* = 233) frequency/median (1st quartile-3rd quartile)	*p*-value (chi squared/Wilcoxon ranksum test)
Baseline characteristics			
Age (months)	28 (4–105)	46 (9–120)	0.129
Female sex (n)	21	109	0.233
Weight (kg)	11.85 (4.50–27.12)	15.95 (7.67–32.68)	0.126
Height (cm)	97 (63–124)	99 (68–138)	0.048
Indicators of disease severity			
PRISM score at PICU admission	14 (8–19)	11 (7–17)	0.155
PIM II score at PICU admission	5 (2–10)	2 (1–7)	0.034
Clinical parameters			
SBP (mmHg)	80.5 (70–94.75)	82 (70–94)	0.763
HR (bpm)	162 (138–180.8)	151 (133–175)	0.947
CVP (mmHg)	11 (8–15.5)	13 (10–16)	0.178
Lactate (mmol/L)	1.8 (1.4–2.77)	2 (1.2–3.8)	0.513
Respiratory frequency (per minute)	25 (15–40)	22 (15–35)	0.973
SpO_2_ (%)	95 (89.25–97)	96 (94–98)	0.015
Urinary excretion (L per day)	3.34 (1.62–4.49)	2.83 (1.88–4.43)	0.582
Core temperature (°C)	39.15 (38.6–39.32)	38.7 (38.2–39)	< 0.001
Blood gases/ laboratory parameters			
pH	7.41 (7.33–7.46)	7.39 (7.32–7.45)	0.388
pCO_2_ (mmHg)	42 (38–50.25)	41 (36–47)	0.433
HCO_3_^−^ (mmol/L)	25 (23–27.25)	24 (22–27)	0.048
Leucocyte count (× 10^9^/L)	11.9 (5.05–18.5)	13.55 (6.6–18.2)	0.593
Hb (g/dL)	10.9 (9.5–12.8)	10.8 (9.5–12.6)	0.331
Platelet count (× 10^9^/L)	112 (44–288.5)	169.5 (110.8–239.8)	0.023
INR	1.42 (1.21–1.67)	1.33 (1.2–1.62)	0.28
PTT (sec)	39.5 (34–50)	35 (31–44.75)	0.013
Fibrinogen (μmol/L)	3.24 (2.31–4.08)	2.6 (1.75–3.8)	0.018
d-dimer (ng/mL)	5008 (2328–11,240)	2733 (1224–5726)	0.001
CRP (mg/L)	48 (30–85.5)	33.5 (12.25–72)	0.009
IL-6 (ng/L)	118 (40–412)	52 (22–122.5)	0.001
PCT (μg/L)	2.55 (0.48–8.45)	1 (0.3–4.17)	0.020
AST (U/L)	75 (34.5–146.5)	73.5 (40–182.5)	0.388
ALT (U/L)	43.5 (16.25–93.5)	32 (18–71.5)	0.295
Phosphate (mmol/L)	1.54 (1.24–1.86)	1.64 (1.27–2.02)	0.238
Creatinine (μmol/L)	33.5 (26.25–61.5)	44.5 (31–65)	0.079
Urea (mmol/L)	7.55 (3.92–11.25)	6.2 (4–10.5)	0.285
Technical ICU support			
Mechanical ventilation (n)	55 (98%)	221 (95%)	0.474
Central venous catheter (n)	43 (77%)	201 (86%)	0.099
Number of peripheral IV cannulas	2 (1–2)	2 (1–2)	0.972
In-line filter application (allocation to interventional group in NCT00209768; ClinicalTrials.gov number)	24 (43%)	102 (44%)	1
Medical/ surgical treatment			
Antibiotics (n)	49 (88%)	195 (84%)	0.545
Steroids (n)	17 (30%)	45 (19%)	0.101
Catecholamines (n)	24 (43%)	132 (57%)	0.074
FiO_2_	0.3 (0.25–0.5)	0.35 (0.24–0.5)	0.806
Surgery before PICU admission (n)	40 (71%)	171 (73%)	0.741
Sepsis/ SIRS related factors			
Length of PICU stay until onset of SIRS/ sepsis (days)	15 (6–41)	2 (1–9)	< 0.001
Cumulative sepsis or SIRS episodes (n)			< 0.001
0	31	199	
1	14	24	
2	7	6	
3	2	4	
4	2	0	
Total SIRS episodes (n)			0.003
0	40	203	
1	11	23	
2	4	6	
3	1	1	
Total sepsis episodes (n)			< 0.001
0	43	223	
1	9	10	
2	2	0	
3	1	0	
4	1	0	

*ALT* alanine transaminase, *AST* aspartate transaminase, *CRP* C-reactive protein, *CVP* central venous pressure, *FiO*_*2*_ fraction of inspired oxygen, *Hb* hemoglobin, *HCO*_*3*_^*−*^ bicarbonate, *HR* heart rate, *ICU* intensive care unit, *IL-6* interleukin 6, *INR* international normalized ratio, *pCO*_*2*_ partial pressure of carbon dioxide, *PCT* procalcitonin, *PTT* partial thromboplastin time, *SBP* systolic blood pressure, *SIRS* systemic inflammatory response syndrome, *SpO*_*2*_ oxygen saturation from pulse oximetry

### Model development

**Fig. 2:**
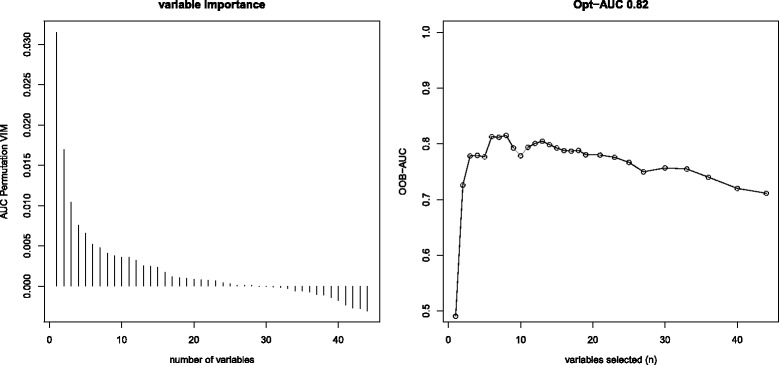
Graphical illustration of the backward variable selection process based on the out-of-bag area under the curve (OOB-AUC). **Left panel:** Area under the curve (AUC) based permutation variable importance measure (VIM) ordered by importance of included variable; the VIM is a proxy for the importance of the variable for correct outcome prediction, but has not the same meaning as classic influence measures based on distributional statistics (like effect sizes (e.g. Odds Ratios) or *p* values). **Right panel:** Areas under the curve by number of included predictor variables (as determined by out-of-bag area under the curve (OOB-AUC) procedure). Corresponding variables can be found in Additional file 1: Table S3

After the dataset was time-split, 130 non-infectious SIRS and 24 sepsis episodes were assigned to the training dataset, while validation was performed on 103 non-infectious SIRS and 32 sepsis cases. Variable selection by a backward selection process in the training dataset showed increasing OOB-AUCs until eight variables were left in the model and decreased afterwards (Fig. 2, Additional file 1: Table S3).

A model including four clinical parameters (length of PICU stay until onset of non-infectious SIRS/sepsis, presence of a central line, core temperature, cumulative number of sepsis and non-infectious SIRS episodes prior to diagnosis) as well as four laboratory parameters (IL-6, platelet count, PCT, CRP) was identified as the best model showing an out-of-bag area under the curve (OOB-AUC) of 0.82 (Fig. 2, Table 2). Analysis of variable importance measures suggested that length of current PICU stay until onset of non-infectious SIRS/sepsis and IL-6 were the most important predictors in our RF approach (Table 2).

**Table 2:** Variables selected for the diagnostic model in the training dataset and their importance

Variable	Variable importance measure^a^
Length of PICU stay until onset of non-infectious SIRS/sepsis	0.031
Interleukin-6	0.017
Platelet count	0.010
Procalcitonin	0.008
Cumulative sepsis or non-infectious SIRS episodes (n)	0.007
Core temperature	0.005
C-reactive protein	0.005
Central venous catheter	0.004

^a^Variable importance measures are a proxy for the importance of the variable for correct outcome prediction, but have not the same meaning as classic influence measures based on distributional statistics (like effect sizes (e.g. Odds Ratios) or *p* values)

### Model performance

The developed prediction model was then applied to the validation dataset reaching a moderate diagnostic accuracy with an AUC of 0.78 (95% CI: 0.70–0.87). When requesting that all sepsis cases were classified as such (correct classification rate of 100% (95% CI: 87–100%)), 28% (95% CI: 20–38%) of non-infectious SIRS episodes were classified correctly. If aiming at the best overall performance as defined by the Youden index, 61% (95% CI: 51–70%) of non-infectious SIRS cases and 84% (95% CI: 66–94%) of sepsis cases could be identified as such.

### Comparison of RF approach to other proposed diagnostic tests

**Fig. 3:**
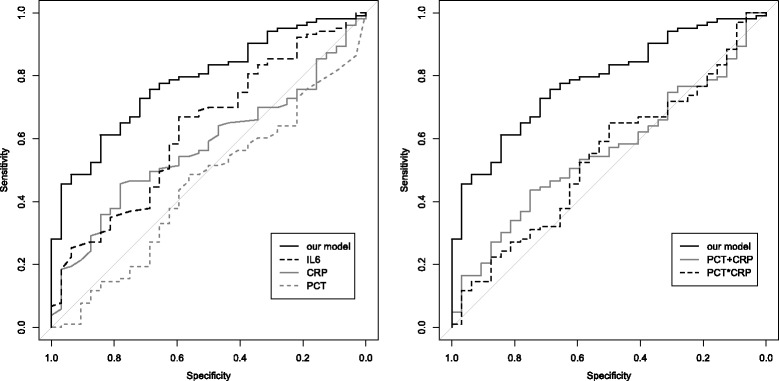
ROC analysis comparing the diagnostic performance of the developed model against previously proposed biomarkers. **Left panel:** The ROC curve of our proposed model (solid black line; AUC: 0.78; 95% CI: 0.70–0.87) was compared against previously proposed single biomarkers in the test data set. C-reactive protein (CRP, solid grey line; AUC = 0.57; 95% CI: 0.47–0.68), interleukin-6 (IL-6, dot-dashed black line; AUC = 0.63; 95% CI: 0.52–0.74) and procalcitonin (PCT, dashed grey line; AUC = 0.55; 95% CI: 0.34–0.56). Specificity represents the correct identification of sepsis, sensitivity the correct identification of SIRS cases. **Right Panel:** The ROC curve of our proposed model (solid black line; AUC: 0.78; 95% CI: 0.70–0.87) was compared against previously proposed combinations of biomarkers. CRP and PCT based on a logistic regression model allowing (dot-dashed black line; AUC = 0.54; 95% CI: 0.43–0.65) and not allowing for interaction (solid grey line; AUC = 0.56; 95% CI: 0.45–0.66). Specificity represents the correct identification of sepsis, sensitivity the correct identification of SIRS cases

Previously proposed markers for the differentiation of non-infectious SIRS and sepsis such as CRP (AUC = 0.57; 95% CI: 0.47–0.68), IL-6 (AUC = 0.63; 95% CI: 0.52–0.74) and PCT (AUC = 0.55; 95% CI: 0.34–0.56) performed worse than the model developed in the RF approach when applied to the validation dataset. Combining CRP and PCT (as proposed by Han et al. in a non-validated study [14]) provided similar accuracy values as the application of single biomarkers (AUC = 0.56; 95% CI: 0.45–0.66 without allowing for interaction; AUC = 0.54; 95% CI: 0.43–0.65 with allowing for interaction, Fig. 3).

### Sensitivity analyses

Three-fold cross-validation showed an average AUC of 0.75, confirming the results of the time-split validation approach. Variation of the RF mtry parameter did not affect accuracy measures (AUCs ranging from 0.72 to 0.84, see Additional file 1: Figure S1). Restriction of the study population to one episode per patient, again, did not have a relevant effect on study results. By using the entire dataset for model development and assessment of performance at the same time, an apparent AUC of 0.98 could be calculated, which overestimates the true predictive performance considerably (see Additional file 1: Figure S2).

## Discussion

In this study, we developed a diagnostic model for the differentiation of sepsis and non-infectious SIRS in critically ill children based on routinely available data. Our developed model was superior to several other previously proposed tests or biomarkers, and could potentially reduce antibiotic treatment by 30% in non-infectious SIRS cases. A combination of 8 out of more than 40 clinical and laboratory parameters was identified as relevant predictors. Some of the identified variables like PCT, CRP and IL-6 have been proposed before as markers for the differentiation between non-infectious SIRS and sepsis [9, 11]; others have not yet been described. These comprise laboratory parameters like platelet count and indicators of disease severity like presence of a central venous line or core temperature. Length of current PICU stay until onset of non-infectious SIRS/sepsis was identified as the most relevant predictor. This can be explained by the fact that most non-infectious SIRS episodes occur early after surgery or trauma and thus early after admission to PICU. In contrast, the risk of sepsis increases with length of stay on PICU.

Previously proposed markers for the differentiation of non-infectious SIRS and sepsis in adults like CRP, IL-6, and PCT performed only slightly better than chance and considerably worse than the model developed in the RF approach, when applied to our data. Even a combination of CRP and PCT (using the same model building approaches as proposed before in a study focusing at a differentiation in the 48 h after disease onset [14]) did not improve their diagnostic accuracy. This emphasizes clearly that not only panels or combinations of biomarkers, but also the additional implementation of clinical parameters as predictors is important when aiming at an improvement of the diagnostic accuracy for the differentiation of sepsis and non-infectious SIRS. Since our study was the first one to take into account all routinely available clinical and laboratory data, it provides an innovative diagnostic approach for sepsis identification which can easily be applied into clinical practice.

One major advantage of our approach is that all relevant information can be entered directly in the model and no further clinical judgement (e.g. on if the SIRS episode happens early or late after admission) needs to be performed. Once an episode of SIRS is identified (e.g. by using a computer-based clinical decision support system implemented in an intensive care unit or by a clinician) and the question arises whether the episode is due to an infection or not, the physician would enter the current values for the eight parameters of our model to an web-based interface (in which the Random Forest construct can be stored), and would promptly receive a decision about if the episode is of infectious origin or not and if antibiotic treatment is necessary. Moreover, probabilities would be given on how likely it is that the episode can be classified as non-infectious SIRS or sepsis. To diminish the risk of mistreatment in septic cases, an episode would only be classified as non-infectious if the model predicts this with 100% probability. Since all of this could happen in routine practice in real-time, even days before microbiological results are expected, treatment initiation could be already triggered by the model results.

### Strengths

Our study has several major strengths. First, the dataset used for our study was very well characterized having been run through various plausibility and quality checks, not the least for the outcome definitions of non-infectious SIRS and sepsis; moreover, it was sufficiently large for the applied analysis strategy allowing time-split validation and accounting for age differences in predictor measures by using age-specific reference values. Moreover, the methodological concept applied to this analysis took advantage of modern machine learning algorithms, developed particularly for situations with many weak predictors as present in our dataset. In contrast to previous studies in the field we rigorously applied the TRIPOD guideline which has become a requirement for high-quality studies in the field of prediction modelling [27]. By combining our purely data-driven approach with rigorously performed validation techniques, we were able to provide a realistic view on the maximum diagnostic accuracy for differentiation of pediatric non-infectious SIRS and sepsis associated with routinely available information. Several previous studies barely mentioned validation processes, so that overfitting and thus overestimation of model performance is very likely [11, 14]. If we did not incorporate validation techniques in our analysis, we got an AUC of 0.98 resulting in an almost perfect discrimination between SIRS and sepsis. In contrast to the model presented in our study, such a model would perform much worse on a new unrelated dataset and would thus not be generalizable. Some of the variables included in our predictive model have not been described previously as strong univariable predictors of the discrimination of non-infectious SIRS and sepsis. The strength of our methodological approach is that it combines their predictive abilities in a non-linear way allowing for hierarchical interactions of the predictors, so that the weaknesses of single predictors in specific situations can be counteracted by other variables in the model.

### Limitations

Our study has several limitations. The data used to develop the prediction model has not been collected for this specific aim. Although secondary data analyses are sometimes associated with severe limitations, the use of the data from a large-sized randomized controlled trial enabled us to combine the advantage of readily available and validated real-life data generated during routine management of a pediatric ICU with the strength of double-validated and blinded outcome definitions of sepsis and non-infectious SIRS. Moreover, no sample size calculation with respect to the discrimination of non-infectious SIRS and sepsis could be performed. The effective sample size of the data has to be regarded as relatively small in the light of the complexity surrounding the subject treated with. However, our dataset represents to our knowledge the largest study on pediatric non-infectious SIRS and sepsis. Moreover, our sensitivity analyses showed that the developed model and its accuracy remained stable over different validation approaches reassuring that the sample size was still large enough for deriving stable estimates.

Though carefully validated, it is not clear if the model can easily be applied to PICUs with standards different from the tertiary-care hospital in which this study was performed. Non-infectious SIRS and sepsis should be diagnosed using the same consensus criteria [1, 3]; predictors being part of the final diagnostic model should be measured in a similar way. Moreover, the generalizability of the model could be impacted by the fact, that we included patients with and without in-line filter treatment [16], even though the original RCT showed that application of in-line filters decreased the risk for non-infectious SIRS. However, the inclusion of all patients led to a more realistic estimate of the diagnostic accuracy of our model when applied to PICUs with differing treatment standards and varying SIRS and sepsis rates, hence possibly facilitating generalizability. Sensitivity analyses restricted to the control group of the RCT showed results compatible to the main analyses.

Nevertheless, external validation of the proposed model in a dataset not related to the present one is necessary to confirm the generalizability of our results.

The data used for this analysis have been collected between 2005 and 2008 so that current treatment practices might not necessarily be reflected. However, since we used pre-treatment parameter values (at least concerning SIRS/sepsis) the risk of a systematic bias by calendar time can be considered as small. In order to avoid a selection bias towards cases occurring early during PICU stay, we used more than one episode per patient for the main analysis. With this approach we might have underestimated the total variability of our dataset and thus might have overestimated the diagnostic accuracy of the model. However, in a sensitivity analysis with only one randomly selected episode per patient we got virtually unchanged results showing that no bias was introduced by our approach.

One general limitation of the RF approach is that it does not allow direct inference on the role of specific predictors like e.g. classic multivariable model building approaches like logistic regression models; it is thus often described as a “black box” since it cannot be used e.g. to develop scores which can be applied with pen and paper but must be run in its original form as a software application to get predictions for new patients. However, variable importance measures can give some information about which variables are most important for discrimination and need to be assessed in order to be able to classify a patient according to the RF based model. While most of the variables included in the final model are routinely available in most ICUs on a daily base, IL-6 and PCT might not which is a potential limitation of our model. In the past years, a new sepsis definition for adult patients was developed [4] which is no longer based on SIRS criteria and might have an impact on future pediatric sepsis definitions [30].

## Conclusions

We have developed and validated for the first time a diagnostic model for the differentiation of non-infectious SIRS and sepsis in critically ill children. It used an innovative methodological approach and identified a combination of eight clinical and laboratory parameters as relevant predictors. The diagnostic accuracy of our model in a validation sample was superior to previously proposed tests for the differentiation of non-infectious SIRS and sepsis when applied to the same dataset. The model allows early recognition of all sepsis cases (correct classification rate of 100%) and could potentially reduce antibiotic use by 30% in non-infectious SIRS cases. All patients in our study were treated with antibiotics at some point during their episode, which underlines the clinical relevance of the proposed reduction in antibiotic treatment for patients with non-infectious SIRS. External validation of our model in an unrelated dataset is necessary to confirm the generalizability of the proposed approach across populations and treatment standards.

## Additional file

Additional file 1: **Table S1**: Overview of all sepsis cases with site of infection and relevant corresponding infectiological data. **Table S2**: Systematic Overview of the Predictors used in the Analysis. **Table S3**: Overview of all models in the backward selection procedure. **Methods S1**: Detailed description and explanation of data analysis approach. **Code S1**: R code for the main analysis. **Figure S1**: AUCs of the time-split approach with different mtry parameter. **Figure S2**: ROC analysis without validation procedure (“Apparent Performance”). (DOCX 81 kb)

## Abbreviations

AUC: Area under the curve
CRP: C-reactive protein
IL-6: Interleukin-6
OOB: Out-of-bag
PCT: Procalcitonin
PICU: Pediatric intensive care unit
RCT: Randomized controlled trial
RF: Random Forrest
SIRS: Systemic inflammatory response syndrome
