# Supplementary material for: Development and validation of a diagnostic model for early differentiation of sepsis and non-infectious SIRS in critically ill children - a data-driven approach using machine-learning algorithms
Source: BMC Pediatr. 2018 Mar 15;18:112. doi: 10.1186/s12887-018-1082-2 (PMC5853156; doi:10.1186/s12887-018-1082-2)
Supplement: Supplementary file 1 — Table S1: Overview of all sepsis cases with site of infection and relevant corresponding infectiological data. Table S2: Systematic Overview of the Predictors used in the Analysis. Table S3: Overview of all models in the backward selection procedure. Methods S1: Detailed description and explanation of data analysis approach. Code S1: R code for the main analysis. Figure S1: AUCs of the time-split approach with different mtry parameter. Figure S2: ROC analysis without validation procedure (“Apparent Performance”). (DOCX 81 kb) [file 12887_2018_1082_MOESM1_ESM.docx]

**Additional files**

p. 2-4 sTable 1: Overview of all sepsis cases with site of infection and relevant corresponding infectiological data

p. 5-8 sTable 2: Systematic Overview of the Predictors used in the Analysis

p. 9 sTable 3: Overview of all models in the backward selection procedure

p. 10-12 sMethods 1: Detailed description and explanation of data analysis approach

p. 13-16 sCode 1: R code for the main analysis

p. 17 sFigure 1: AUCs of the time-split approach with different mtry parameter

p. 18 sFigure 2: ROC analysis without validation procedure (“Apparent Performance”)

**sTable 1: Overview of all sepsis cases with side of infection and relevant corresponding infectiological data**

| **episode** | **primary site of infection** | **infectiological data** |
| --- | --- | --- |
| 1 | primary meningitis | liquor leucocytosis, no organism cultured after empiric antibiotic treatment |
| 2 | ventilator-associated pneumonia | microbiologically confirmed in cultures of a bronchoalveolar lavage sample |
| 3 | catheter-related bloodstream infection | catheter tip culture, positive paired central and peripheral blood cultures, all growing the same organism |
| 4 | ventilator-associated pneumonia | microbiologically confirmed in cultures of a bronchoalveolar lavage sample |
| 5 | secondary peritonitis | isolation of microbial pathogens found in the peritoneum following gastrointestinal perforation |
| 6 | catheter-related bloodstream infection | catheter tip culture, positive paired central and peripheral blood cultures, all growing the same organism |
| 7 | catheter-related bloodstream infection | catheter tip culture, positive paired central and peripheral blood cultures, all growing the same organism |
| 8 | peritonitis with secondary bloodstream infection | microbiologically confirmed in direct surgical collection and blood cultures, all growing the same organism |
| 9 | peritonitis with secondary bloodstream infection | microbiologically confirmed in direct surgical collection and blood cultures, all growing the same organism |
| 10 | pneumonia and bloodstream infection | confirmed in bronchoalveolar lavage sample and blood sample, immunocompromised patient |
| 11 | primary bloodstream infection | microbiologically confirmed in blood cultures |
| 12 | primary meningitis | Waterhouse-Friderichsen syndrome |
| 13 | primary meningitis | liquor leucocytosis, no organism cultured after empiric antibiotic treatment |
| 14 | ventilator-associated pneumonia | microbiologically confirmed in cultures of a bronchoalveolar lavage sample |
| 15 | blood stream infection | confirmed in blood sample, immunocompromised patient |
| 16 | biliary tract infection | microbiologically confirmed in direct surgical collection of samples of the biliary tract |
| 17 | catheter-related bloodstream infection | catheter tip culture, positive paired central and peripheral blood cultures, all growing the same organism |
| 18 | catheter-related bloodstream infection | catheter tip culture, positive paired central and peripheral blood cultures, all growing the same organism |
| 19 | catheter-related bloodstream infection | catheter tip culture, positive paired central and peripheral blood cultures, all growing the same organism |
| 20 | primary meningitis | liquor leucocytosis, no organism cultured after empiric antibiotic treatment |
| 21 | catheter-related bloodstream infection | catheter tip culture, positive paired central and peripheral blood cultures, all growing the same organism |
| 22 | urosepsis in noncatheterized patients | pyuria, organism isolated from urin culture in neonate with rectovesical fistula and evidence of infection on ultrasound |
| 23 | ventilator-associated pneumonia with secondary bloodstream infection | microbiologically confirmed in cultures of a bronchoalveolar lavage sample and blood cultures, all growing the same organism |
| 24 | urosepsis in catheterized patients | pyuria, organism isolated from urin culture |
| 25 | pneumonia | confirmed in bronchoalveolar lavage sample |
| 26 | ventilator-associated pneumonia | microbiologically confirmed in cultures of a bronchoalveolar lavage sample |
| 27 | urosepsis in catheterized patients | pyuria, organisms seen on Gram stain of unspun urine |
| 28 | catheter-related bloodstream infection | positive paired central and peripheral blood cultures, all growing the same organism |
| 29 | biliary tract infection | microbiologically confirmed in percutaneous collection of samples of the biliary tract |
| 30 | peritoneal dialysis-related peritonitis | microbiologically confirmed in cultures of peritoneal fluid sample |
| 31 | secondary peritonitis | isolation of microbial pathogens found in the peritoneum following gastrointestinal perforation |
| 32 | primary peritonitis | microbiologically confirmed in cultures of peritoneal fluid sample |
| 33 | primary peritonitis | microbiologically confirmed in cultures of peritoneal fluid sample |
| 34 | catheter-related bloodstream infection | positive paired central and peripheral blood cultures, all growing the same organism |
| 35 | peritonitis with secondary bloodstream infection | microbiologically confirmed in direct surgical collection and blood cultures, all growing the same organism |
| 36 | biliary tract infection | microbiologically confirmed in percutaneous collection of samples of the biliary tract |
| 37 | catheter-related bloodstream infection | positive paired central and peripheral blood cultures, all growing the same organism |
| 38 | secondary peritonitis | isolation of microbial pathogens found in the peritoneum following gastrointestinal perforation |
| 39 | catheter-related bloodstream infection | catheter tip culture, positive peripheral blood cultures, all growing the same organism |
| 40 | secondary peritonitis | isolation of microbial pathogens found in the peritoneum following gastrointestinal perforation |
| 41 | primary peritonitis | microbiologically confirmed in cultures of peritoneal fluid sample |
| 42 | catheter-related bloodstream infection | positive paired central and peripheral blood cultures, all growing the same organism |
| 43 | primary bloodstream infection | confirmed in blood sample, immunocompromised patient |
| 44 | primary meningitis | liquor leucocytosis, no organism cultured after empiric antibiotic treatment |
| 45 | catheter-related bloodstream infection | catheter tip culture, positive peripheral blood cultures, all growing the same organism |
| 46 | primary bloodstream infection | microbiologically confirmed in blood cultures |
| 47 | biliary tract infection with secondary blood stream infection | microbiologically confirmed in blood cultures |
| 48 | catheter-related bloodstream infection | catheter tip culture, positive paired central and peripheral blood cultures, all growing the same organism |
| 49 | bloodstream infection | confirmed in blood sample, immunocompromised patient |
| 50 | biliary tract infection | microbiologically confirmed in direct surgical collection of samples of the biliary tract |
| 51 | peritonitis with secondary bloodstream infection | microbiologically confirmed in blood cultures |
| 52 | ventilator-associated pneumonia with secondary bloodstream infection | microbiologically confirmed in blood cultures |
| 53 | ventilator-associated pneumonia with secondary bloodstream infection | microbiologically confirmed in blood cultures |
| 54 | ventilator-associated pneumonia with secondary bloodstream infection | microbiologically confirmed in cultures of a bronchoalveolar lavage sample and blood cultures, all growing the same organism |
| 55 | ventilator-associated pneumonia with secondary bloodstream infection | microbiologically confirmed in cultures of a bronchoalveolar lavage sample and blood cultures, all growing the same organism |
| 56 | catheter-related bloodstream infection | catheter tip culture, positive paired central and peripheral blood cultures, all growing the same organism |

**sTable 2: Systematic Overview of the Predictors used in the Analysis**

| **description of predictor variable** | **type of predictor variable** | **levels/units** | **value representative for^*^** | **source of reference values used for age standardization^**^** |
| --- | --- | --- | --- | --- |
| 1. age | quantitative | days | day of diagnosis | - |
| 1. sex | qualitative | 0 = female, 1 = male | entire study period | - |
| 1. weight at admission | quantitative | kg | entire study period | KiGGS [1] |
| 1. height at admission | quantitative | cm | entire study period | KiGGS [1] |
| 1. surgery previous to PICU admission | qualitative | 0 = no, 1 = yes | entire study period | - |
| 1. PRISM score at PICU admission | quantitative | score | entire study period | - |
| 1. PIM II at PICU admission | quantitative | score | entire study period | - |
| 1. length of PICU stay until onset of SIRS/ sepsis | quantitative | days | day of diagnosis | - |
| 1. central venous catheter | qualitative | 0 = no, 1 = yes | day of diagnosis | - |
| 1. mechanical ventilation | qualitative | 0 = no, 1 = yes | day of diagnosis | - |
| 1. fraction of inspired oxygen (fiO_2_)*** | quantitative | % | day of diagnosis | - |
| 1. respiratory frequency | quantitative | breaths per minute | day of diagnosis | Fleming et. al [2] |
| 1. pH (blood gas) | quantitative | - | day of diagnosis | Gregory and Andropoulos [3] |
| 1. partial pressure of carbon dioxide (pCO_2_) | quantitative | mmHg | day of diagnosis | Gregory and Andropoulos [3] |
| 1. bicarbonate (HCO_3_^-^) | quantitative | mmol/L | day of diagnosis | - |
| 1. oxygen saturation (SO_2_) | quantitative | % | day of diagnosis | - |
| 1. central venous oxygen saturation (ScvO2) | quantitative | % | day of diagnosis | - |
| 1. lactate | quantitative | mmol/L | day of diagnosis | Mayo Medical Laboratories [4] |
| 1. central venous pressure (CVP) | quantitative | mmHg | day of diagnosis | - |
| 1. systolic blood pressure (SBP) | quantitative | mmHg | day of diagnosis | Gebara [5] |
| 1. heart rate (HR) | quantitative | bpm | day of diagnosis | Fleming et. al [2] |
| 1. core temperature | quantitative | °C | day of diagnosis | - |
| 1. aspartate transaminase (AST), serum | quantitative | U/L | day of diagnosis | Gregory and Andropoulos [3] |
| **description of predictor variable** | **type of predictor variable** | **levels/units** | **value representative for^*^** | **source of reference values used for age standardization^**^** |
| 1. alanine transaminase (ALT), serum | quantitative | U/L | day of diagnosis | Gregory and Andropoulos [3] |
| 1. glutamate dehydrogenase (GLDH) | quantitative | U/L | day of diagnosis | - |
| 1. phosphate, serum (PO_4_^3-^) | quantitative | mmol/L | day of diagnosis | Institute for Clinical Chemistry, MHH [6] |
| 1. creatinine, serum | quantitative | μmol/L | day of diagnosis | Institute for Clinical Chemistry, MHH [6] |
| 1. urea | quantitative | mmol/L | day of diagnosis | - |
| 1. urinary excretion | quantitative | L per day | day of diagnosis | - |
| 1. C-reactive protein (CRP) | quantitative | mg/L | day of diagnosis | - |
| 1. procalcitonin, serum (PCT) | quantitative | μg/L | day of diagnosis | -**** |
| 1. interleukin 6 (IL-6) | quantitative | ng/L | day of diagnosis | - |
| 1. hemoglobin (Hb) | quantitative | g/dL | day of diagnosis | Gregory and Andropoulos [3] |
| 1. leucocyte count | quantitative | x 10^9^/L | day of diagnosis | IPSCC [7]***** |
| 1. platelet count | quantitative | x 10^9^/L | day of diagnosis | - |
| 1. fibrinogen | quantitative | μmol/L | day of diagnosis | - |
| 1. D-dimer | quantitative | ng/mL | day of diagnosis | - |
| 1. partial thromboplastin time (PTT) | quantitative | seconds | day of diagnosis | - |
| 1. number of peripheral IV cannulas | quantitative | - | day of diagnosis | - |
| 1. international normalized ratio (INR) | quantitative | ratio | day of diagnosis | - |
| 1. treatment with antibiotics****** | qualitative | 0 = no, 1= yes | day of diagnosis | - |
| 1. treatment with steroids******* | qualitative | 0 = no, 1= yes | day of diagnosis | - |
| 1. treatment with catecholamines | qualitative | 0 = no, 1= yes | day of diagnosis | - |
| 1. cumulative sepsis or SIRS episodes (n) | quantitative | - | time period before and day of diagnosis | - |
| 1. total SIRS episodes (n) | quantitative | - | time period before and day of diagnosis | - |
| **description of predictor variable** | **type of predictor variable** | **levels/units** | **value representative for^*^** | **source of reference values used for age standardization^**^** |
| 1. total sepsis episodes (n) | quantitative | - | time period before and day of diagnosis | - |

^*^If a predictor was measured more than once a day, the most abnormal value was recorded in the dataset. The most abnormal one was judged from a clinical point of view.

In the case of predictors being part of the blood gas analysis, all values belonging to the most abnormal blood gas analysis were taken. Subject of clinical judgement thus wasn´t one single predictor but the entire blood gas analysis.

^**^Age standardization was performed by dividing the individual value by the median reference value for the specific age group. If percentiles were given this corresponded to the 50^th^ percentile; if a reference range with a lower and upper limit was given, this corresponded to the arithmetic mean of the both. In the case of different reference values for boys and girls they were applied accordingly.

***In the absence of mechanical ventilation, we imputed 0.21 for any missing value which represents the fraction of oxygen in the ambient air.

****Even if it is known that procalcitonin rises in newborns shortly after birth, it was not necessary to perform an adjustment since there was no newborn with a measured procalcitonin value in our dataset.

*****Since no lower limit for children aged 0 days to 1 week was given, we used the lower limit of the adjacent age group (aged 1 week to 1 month) to calculate the arithmetic mean used for adjustment.

******At the day the start of the episode was defined

*******Steroids here refer to steroids possibly affecting a change in leucocyte count (not hydrocortisone).

Since the rationale behind any kind of adjustment is to diminish the influence of factors which would inhibit the performance of a statistical analysis leading to reliable results, sources for reference values were selected in the following manner:

1. Laboratory predictors
   1. Knowing that laboratory test results differ dependent on the laboratory analyzing the samples, we tried to use reference values provided by the laboratory which analyzed the samples taken in the conducted trial. Thus, in the first instance, we aimed to use reference values provided by the Institute for Clinical Chemistry of Hanover Medical School. If there were none available, we applied reference values obtained via a literature search.
   2. For leucocytes we needed to apply the reference values of the adjacent age group since there were no reference values available for some age groups.
2. Predictors other than laboratory predictors
   1. For predictors other than laboratory predictors we tried to use the latest reference values available. We took into account that some predictors, such as weight and height, might be dependent on genetic factors and preferred therefore to use reference values derived from the German population.

References

[1] Robert Koch Institut, “Referenzperzentile für anthropometrische Maßzahlen und Blutdruck aus der Studie zur Gesundheit von Kindern und Jugendlichen in Deutschland (KiGGS).” .

[2] S. Fleming, M. Thompson, R. Stevens, C. Heneghan, A. Plüddemann, I. Maconochie, L. Tarassenko, and D. Mant, “Normal ranges of heart rate and respiratory rate in children from birth to 18 years: a systematic review of observational studies,” *Lancet*, vol. 377, no. 9770, pp. 1011–1018, Mar. 2011.

[3] “Wiley: Gregory’s Pediatric Anesthesia, 5th Edition - George A. Gregory, Dean B. Andropoulos.” [Online]. Available: http://eu.wiley.com/WileyCDA/WileyTitle/productCd-EHEP002663.html. [Accessed: 19-Oct-2015].

[4] “Pediatric Test Reference Values - Mayo Medical Laboratories.” [Online]. Available: http://www.mayomedicallaboratories.com/test-info/pediatric/refvalues/index.html. [Accessed: 19-Oct-2015].

[5] B. M. Gebara, “Values for systolic blood pressure,” *Pediatr. Crit. Care Med. J. Soc. Crit. Care Med. World Fed. Pediatr. Intensive Crit. Care Soc.*, vol. 6, no. 4, p. 500; author reply 500–501, Jul. 2005.

[6] Institute for Clinical Chemistry, Hanover Medical School, “Blaues Heft.” .

[7] B. Goldstein, B. Giroir, A. Randolph, and International Consensus Conference on Pediatric Sepsis, “International pediatric sepsis consensus conference: definitions for sepsis and organ dysfunction in pediatrics,” *Pediatr. Crit. Care Med. J. Soc. Crit. Care Med. World Fed. Pediatr. Intensive Crit. Care Soc.*, vol. 6, no. 1, pp. 2–8, Jan. 2005.

[8] T. Jack, M. Boehne, B. E. Brent, L. Hoy, H. Köditz, A. Wessel, and M. Sasse, “In-line filtration reduces severe complications and length of stay on pediatric intensive care unit: a prospective, randomized, controlled trial,” *Intensive Care Med.*, vol. 38, no. 6, pp. 1008–1016, Jun. 2012.

**sTable 3: Overview of all models in the backward selection procedure**

| **number of predictor variables in the model** | **OOB-AUC** | **predictor variables eliminated in next step** |
| --- | --- | --- |
| 44 | 0.711 | phosphate |
|  |  | height |
|  |  | female sex |
|  |  | respiratory frequency |
| 40 | 0.720 | PRISM score at PICU admission |
|  |  | number of peripheral IV cannulas |
|  |  | leucocyte count |
|  |  | age |
| 36 | 0.74 | catecholamines |
|  |  | HR |
|  |  | mechanical ventilation |
| 33 | 0.755 | pCO_2_ |
|  |  | antibiotics |
|  |  | cumulative sepsis episodes |
| 30 | 0.757 | Hb |
|  |  | weight |
|  |  | AST |
| 27 | 0.750 | FiO_2_ |
|  |  | SBP |
| 25 | 0.767 | ALT |
|  |  | surgery before PICU admission |
| 23 | 0.776 | PIM II score at PICU admission |
|  |  | steroids |
| 21 | 0.780 | creatinine |
|  |  | urinary excretion |
| 19 | 0.780 | lactate |
| 18 | 0.788 | SpO_2_ |
| 17 | 0.787 | INR |
| 16 | 0.788 | d-dimer |
| 15 | 0.793 | PTT |
| 14 | 0.798 | pH |
| 13 | 0.805 | CVP |
| 12 | 0.801 | cumulative SIRS epsisodes |
| 11 | 0.794 | HCO_3_- |
| 10 | 0.779 | urea |
| 9 | 0.793 | fibrinogen |
| 8 | 0.815 | central venous catheter |
| 7 | 0.812 | CRP |
| 6 | 0.813 | core temperature |
| 5 | 0.777 | cumulative sepsis or SIRS episodes |
| 4 | 0.779 | PCT |
| 3 | 0.778 | platelet count |
| 2 | 0.726 | IL-6 |
| 1 | 0.490 | length of PICU stay until onset of SIRS/ sepsis |

ALT, alanine transaminase; AST, aspartate transaminase; CRP, C-reactive protein; CVP, central venous pressure; FiO_2_, fraction of inspired oxygen; Hb, hemoglobin; HCO_3_^-^, bicarbonate; HR, heart rate; ICU, intensive care unit; IL-6, interleukin 6; INR, international normalized ratio; pCO_2_, partial pressure of carbon dioxide; PCT, procalcitonin; PTT, partial thromboplastin time; SBP, systolic blood pressure; SIRS, systemic inflammatory response syndrome; SpO_2_, oxygen saturation from pulse oximetry.

**sMethods 1: Detailed description and explanation of data analysis approach**

Random Forests are a so called statistical ensemble method based on tree predictors. The idea behind a statistical ensemble method is to combine different statistical learners to improve prediction accuracy. In Random Forests the statistical learners are decision trees. The trees of the forest are grown based on the same tuning parameters (such as mincriterion, minbucket, minsplit and mtry [see definition below]), but use different subsamples of the data (in the sense of different subsets of the observations). By growing trees to different subsamples of the data, the resulting trees (=statistical learners) differ from each other. In Random Forests, there is, however, another process by which random variation is implemented. At each split (bifurcation, ´subdivision of a branch´) only a randomly drawn subset of all predictor variables is considered. The size of this subset is denoted by the mtry parameter. By introducing variation on both, the level of the observations and the level of the variables, resulting trees are just different enough so that they fulfil the requirements of good ensemble methods.

If classification is the purpose, as it is in our study, an observation (non-infectious SIRS/sepsis episode) in one tree may be predicted as belonging to the one class (e.g. non-infectious SIRS) and in another tree as belonging to the other class (sepsis). Each of the trees ´votes´ (see e.g. [1]) for one of the classes. In the end an observation is predicted as belonging to the class for which it got most votes over all trees.

Since in Random Forests every tree is just fit to a subsample of the data, not every observation is used to construct and train the respective tree. The observations not used to construct a specific tree are for this tree ´out of bag´ (OOB), a term arising from the historic precursor of Random Forests, bagging (bootstrap aggregation). Since these observations were not used in constructing the specific tree (= not used to fit the statistical learner), they are independent of this tree and can be considered as test data for it. Typically, 1/3 of the observations are OOB for every tree which means that every tree is constructed using on average 2/3 of the observations. If a Random Forest is built with 1000 trees (ntree = 1000), for every observation there are approximately 333 trees, which can be used for independent prediction. Thus, a valid estimate of the predictive performance of the entire forest can be obtained by applying the ´most voted strategy´ to these ´OOB-votes´. This estimate is called the OOB estimate of error or OOB-error and is just a misclassification error, so that concepts such as calculating the area under the curve (AUC) via the ROC curve can be performed easily. In analogy to the OOB-error the corresponding AUC is then called OOB-AUC.

Imbalanced datasets are datasets in which the frequency of outcome categories differs. In our case, non-infectious SIRS cases are much more frequent than sepsis cases which can lead to problems in prediction modelling. We therefore used a Variable Importance Measure for initial variable ranking that does not prefer the majority class, namely the AUC-based permutation Variable Importance Measure, and implemented the AUC as model accuracy measure instead of the misclassification error rate based on the most voted strategy (see above and e.g. [1]).

In the past years there were proposed different methods to assess variable importance (so called variable importance measures (VIMs)) for Random Forests. One of the most popular ones is the permutation VIM or error-rate-based permutation VIM which is based on the OOB-error-rate [10]. The AUC-based permutation VIM uses the AUC calculated from the OOB observations of a tree. The permutation VIM is calculated by subtracting the AUC obtained when the values of a predictor are randomly assigned to the different observations (“permuted”, using the mean AUC of e.g. 1000 random permutations) from the AUC obtained when the values are assigned to the correct observation.

We used standard literature recommendations for the number of trees (ntree) and the number of variables used for selection per bifurcation (mtry). For ntree it has been shown exhaustively that once it exceeds a certain number (e.g. 500) its value does no longer affect prediction precision but only computing time [2]. We nevertheless repeated our analysis with larger values for ntree and found, as expected, no relevant differences.

The number of variables selected as potential predictors at each bifurcation (mtry) is more controversially discussed and known to be the most important parameter to set [3]. A standard approach and usually a good choice [3] is to use the square root of the total number of variables in the dataset (also our starting point) while some researchers suggest that values closer to ½ or 2/3 of the total number of variables might yield better results. Others say that a relatively small value can be especially helpful if there is one (ore just a few) strong predictors among many weak or if a large number of variables is expected to be highly correlated [4]. We therefore varied mtry for our sensitivity analyses to all possible values and found virtually no effects on the predictive performance (see sFigure1). This shows that our approach is robust with respect to the most crucial input parameter of Random Forests.

The parameter mincriterion, minbucket and minsplit are particularly inherent to the party package. In a nutshell, the smaller the three parameters, the higher the variance of a tree. If then a forest is grown while these parameters are small, all trees will look more different from each other than they would if the parameters were set to a higher value. This aims at the machine learning principle of achieving a low-bias-high-variance model by averaging (majority vote) over a set of high-variance learners (different looking trees). We used the three parameters exactly as Janitza et. al did [5].

The variable selection process was performed as follows:

Stage 1: Iterative Elimination Process.

1. We built a first RF using all predictor variables and computed its OOB-AUC.
2. We ranked the predictor variables based on the AUC-based permutation VIM.
3. We eliminated (down-rounded) 10 % of the less important predictor variables based on the initial ranking.
4. We built a RF using the reduced set of predictor variables and computed its OOB-AUC.
5. We repeated steps 3 to 4 until the number of remaining predictor variables was equal to one.

Stage 2: Visual Representation of the Elimination Process.

We visualized the elimination process with a curve describing the OOB-AUC value of the different RFs (y-axis) as a function of the number of predictor variables (x-axis).

Stage 3: Selection of the Optimal Set of Predictors.

The optimal set of predictor variables was considered as the one giving rise to the RF with the highest OOB-AUC.

[Annotations:

- This selection procedure differs from the one presented in [1] mainly by using the AUC-based permutation VIM instead of the Mean decreased Gini (MDG) as VIM. The MDG-VIM was shown to be biased to certain types of predictors [5]. The AUC-based permutation VIM outperforms others when class sizes are different, as it is the case in our data.
- To eliminate in every step (down-rounded) 10 % and no other quantity of the predictors is an arbitrary decision. Once the number of variables in the model is smaller than 10, variables are eliminated one by one.
- Tuning parameters of random forest are kept fixed throughout the entire selection procedure. This is crucial to obtain comparable performance measures for different sets of variables. However, neither Calle et. al [1] nor Diaz-Uriarte et. al [3] report how mtry behaves if the number of variables still present becomes smaller than the mtry value prespecified. In our selection procedure it is by default reduced to the number of variables still present .]

References

[1] Calle ML, Urrea V, Boulesteix AL, Malats N. AUC-RF: a new strategy for genomic profiling with random forest. Human heredity. 2011;72:121-32.

[2] Oshiro TM, Perez PS, Baranauskas JA. How Many Trees in a Random Forest? In: Perner P, editor. Machine Learning and Data Mining in Pattern Recognition: 8th International Conference, MLDM 2012, Berlin, Germany, July 13-20, 2012 Proceedings. Berlin, Heidelberg: Springer Berlin Heidelberg; 2012. p. 154-68.

[3] Diaz-Uriarte R, Alvarez de Andres S. Gene selection and classification of microarray data using random forest. BMC bioinformatics. 2006;7:3.

[4] James G, Witten D, Hastie T, Tibshirani R. An Introduction to Statistical Learning: with Applications in R: Springer Publishing Company, Incorporated; 2014.

[5] Janitza S, Strobl C, Boulesteix AL. An AUC-based permutation variable importance measure for random forests. BMC bioinformatics. 2013;14:119.

**sCode 1: R code for the main analysis**

###################################################

## VALIDATION USING A TIME BASED SPLIT-SAMPLE

###################################################

###################################################

library(pROC)

library(party)

library(randomForest)

library(missForest)

#####################################

# 1 Splitting data based on time ####

#####################################

data <- data_expe

data1 <- data[data$year<=2006, ]

sum(as.character(data1$sirs)=="sirs")

sum(as.character(data1$sirs)=="sepsis")

data2 <- data[data$year>2006, ]

sum(as.character(data2$sirs)=="sirs")

sum(as.character(data2$sirs)=="sepsis")

train <- data1

train$year <- NULL

test <- data2

test$year <- NULL

###########################

# 2 First Split-Sample ####

###########################

# 2.1 Missing Imputation using MissForest ####

##############################################

# holding out outcome variable and previous events #

vars_holdout <- c("sirs", "prevsepsis", "prevsirs", "prevevents")

train_holdout <- train[names(train) %in% vars_holdout]

train_imp <- train[names(train) %in% vars_holdout==F]

set.seed(90)

imp <- missForest(train_imp, maxiter = 10, ntree = 100, variablewise = F,

decreasing = FALSE, verbose = TRUE,

mtry = floor(sqrt(ncol(train_imp))), replace = TRUE,

classwt = NULL, cutoff = NULL, strata = NULL,

sampsize = NULL, nodesize = NULL, maxnodes = NULL,

xtrue = NA, parallelize = c("no", "variables", "forests"))

train_imp <- imp$ximp

train_imp <-cbind(train_imp, train_holdout)

train_imp$BE <- NULL

train <- train_imp

# 2.2 Backward Elimination Procedure ####

#########################################

# 2.2.1 Initial Ranking of Predictor Variables ####

m <- round(sqrt((ncol(train_imp))-1))

# Arguments to cforest #

forest_control <- cforest_control(teststat = "quad",

testtype = "Univ",

mincriterion = 0,

minsplit = 0,

minbucket = 0,

ntree = 1000,

replace = T,

trace=T,

mtry=m)

# Forest Construction and Ranking #

set.seed(90)

forest <- cforest(sirs ~ ., data = train,

controls = forest_control)

varimp.AUC <- varimpAUC(forest)

varimp.AUC <- sort(varimp.AUC, decreasing=T)

plot(1:length(varimp.AUC), varimp.AUC, type="h",

main="variable importance", ylab="AUC Permutation VIM",

xlab="number of variables")

# 2.2.2 Backward Elimination ####

# Performing Backward Elimination ####

backward_list <- list(varimp.AUC, varimp.AUC)

back <- varimp.AUC

repeat {

back <- back[-c(length(back):ceiling(length(back)-length(back)*0.1+1))]

backward_list <- c(backward_list, list(back))

if (length(back)==1) break

}

backward_list <- backward_list[-c(1)]

aucs <- rep(0, length(backward_list))

n_vars <- rep(0, length(backward_list))

for (i in 1:length(backward_list)){

n_vars[i] <- length(backward_list[[i]])

train <- train[, names(train) %in% c(names(backward_list[[i]]), "sirs")]

set.seed(90)

forest <- cforest(sirs ~ ., data = train,

controls = forest_control)

probs_both <- predict(forest, OOB=T, type="prob")

probs <- rep(0, nrow(train))

for(j in 1:nrow(train)){

probs[j] <- probs_both[[j]][, 2]

}

probs

roc.oob <- roc(train$sirs, probs)

aucs[i] <- roc.oob$auc[1]

}

# Visual Representation of the Elimination Process ####

plot(n_vars, aucs, ylab="OOB-AUC", xlab="variables selected (n)",

main=paste("Opt-AUC", round(max(aucs), digit=2)), ylim=c(0.5,1))

lines(n_vars, aucs)

# Selection of the best model ####

time_split_elim <- data.frame(aucs, n_vars)

time_split_elim$sub <- seq_len(nrow(time_split_elim))

time_split_opt_model_sub <- time_split_elim$sub[time_split_elim$aucs==max(time_split_elim$aucs)]

if(length(time_split_opt_model_sub)>1){

time_split_opt_model_sub <- max(time_split_opt_model_sub)

}

time_split_opt_model <- backward_list[time_split_opt_model_sub]

############################

# 3 Second-Split-Sample ####

############################

# 3.1 Missing Imputation using MissForest ####

##############################################

# holding out outcome variable and previous events #

vars_holdout <- c("sirs", "prevsepsis", "prevsirs", "prevevents")

test_holdout <- test[names(test) %in% vars_holdout]

test_imp <- test[names(test) %in% vars_holdout==F]

set.seed(90)

imp <- missForest(test_imp, maxiter = 10, ntree = 100, variablewise = F,

decreasing = FALSE, verbose = TRUE,

mtry = floor(sqrt(ncol(test_imp))), replace = TRUE,

classwt = NULL, cutoff = NULL, strata = NULL,

sampsize = NULL, nodesize = NULL, maxnodes = NULL,

xtrue = NA, parallelize = c("no", "variables", "forests"))

test_imp <- imp$ximp

test_imp <-cbind(test_imp, test_holdout)

test_imp$BE <- NULL

test <- test_imp

###################

# 4 Validation ####

###################

# Prediction Train - Test ####

train <- train_imp

train <- train[, names(train) %in% c(names(backward_list[[time_split_opt_model_sub]]), "sirs")]

set.seed(90)

forest <- cforest(sirs ~ ., data = train,

controls = forest_control)

test <- test[, names(test) %in% c(names(backward_list[[time_split_opt_model_sub]]), "sirs")]

a <- predict(forest, newdata=test, type="prob")

probs <- rep(0, nrow(test))

for(j in 1:nrow(test)){

probs[j] <- a[[j]][, 2]

}

time_split_roc <- roc(test$sirs, probs, plot=T)

*Annotations:*

- *Note that for reasons of reproducibility we copied our syntax exactly as we used it to analyze our data. Thus, if this syntax is applied to other data than ours you will have to make some changes to it (e. g. change labeling of the outcome variable (´sirs´), change the threshold for time-splitting (´data1 <- data[data$year<=2006, ]´ and ´data2 <- data[data$year>2006, ]´) and omit ´train_imp$BE <- NULL´ as well as ´test_imp$BE <- NULL´). The latter corresponds to omittance of the base excess (BE), which by clinical expert opinion was not considered as a potential predictor for model development to reduce bias for reasons like multicollinearity, since it nearly reflects the same information as bicarbonate.*
- *Note that when the number of variables during the backward selection process gets smaller than mtry, mtry is reduced to this number of variables still in the model (Warning message: “In model@fit(data, …) : mtry is larger than ninputs, using mtry = inputs”, see also description of backward selection in sMethods1).*

**sFigure 1: AUCs of the time-split approach with different mtry parameter**

Outcome prediction accuracy measured by the AUC (AUCs ranging from 0.72 to 0.84) for different mtry parameters. Prediction was performed as in the main approach using the data of 2005 to 2006 as training and data of 2007 to 2008 as test data.

**sFigure 2: ROC analysis using the entire dataset as training data without validation procedure (“Apparent Performance”)**

Possible outcome prediction accuracy measured by the AUC (AUC = 0.98; 95% CI: 0.97-0.99) if no validation procedure was performed.
